# Papillary Renal Cell Carcinoma Presented With Supraclavicular Lymph Node Metastasis Without Renal Primary Lesion

**DOI:** 10.4021/wjon593w

**Published:** 2013-03-06

**Authors:** Natanong Thamcharoen, Worawit Chaiwiriyawong

**Affiliations:** a Chulabhorn Hospital, Bangkok, Thailand

**Keywords:** Renal cell carcinoma, Unknown primary cancer, Lymph node metastasis, Without primary site

## Abstract

Renal cell carcinoma is a rare cancer in Thailand. Most of the patients present in advanced metastatic stage with identifiable renal mass. In this case report, we presents a case of male patient who manifested with supraclavicular lymph node enlargement and CT scan of chest and abdomen showed multiple sites lymph node metastasis but there was no primary mass detected anywhere. The pathology of supraclavicular lymph node was papillary cell adenocarcinoma. The differential diagnoses were papillary thyroid cancer, gastrointestinal tract carcinoma such as pancreato-biliary cancer, non small cell lung cancer, and renal cancer. Immunohistochemistry result were negative for TTF-1, Thyroglobulin, CD7 and CD20 which ruled out non-small cell lung adenocarcinoma, thyroid cancer and gastrointestinal tract cancer respectively. CD10, Vimentin and RCC were all positive and all are specific for renal cell carcinoma. The diagnosis was renal cell carcinoma, papillary cell type. Sunitinib, a tyrosine kinase inhibitor, is the treatment of choice for renal cell carcinoma since it improves objective response rate and shows longer progression free survival than IFNα.

## Introduction

In Thailand, renal cell carcinoma (RCC) is a rare malignant condition among men and women, accounting for 0.7% of all cancers [1]. It occurs mainly in male 6th - 8th decade of life. The classical triad of the renal cell carcinoma symptoms consists of hematuria, abdominal mass and flank pain. Still, only about 10% of patients presented with triad which defines advanced disease. Approximately half of cases are now detected because of accidental finding of renal mass from imaging. However, the disease is well-known for metastatic ability. The target for metastasis are lungs, bone, lymph node, adrenal gland, brain, liver and contralateral kidney. A quarter of the patients present with advanced disease, including locally invasive or metastatic renal-cell carcinoma but it is very uncommon to present without primary lesion at kidney [2]. In this case report, we will discuss about a renal cell carcinoma patient who presented with supraclavicular lymph node enlargement as first symptoms and no renal mass could be detected.

## Case Report

**Figure 1:**
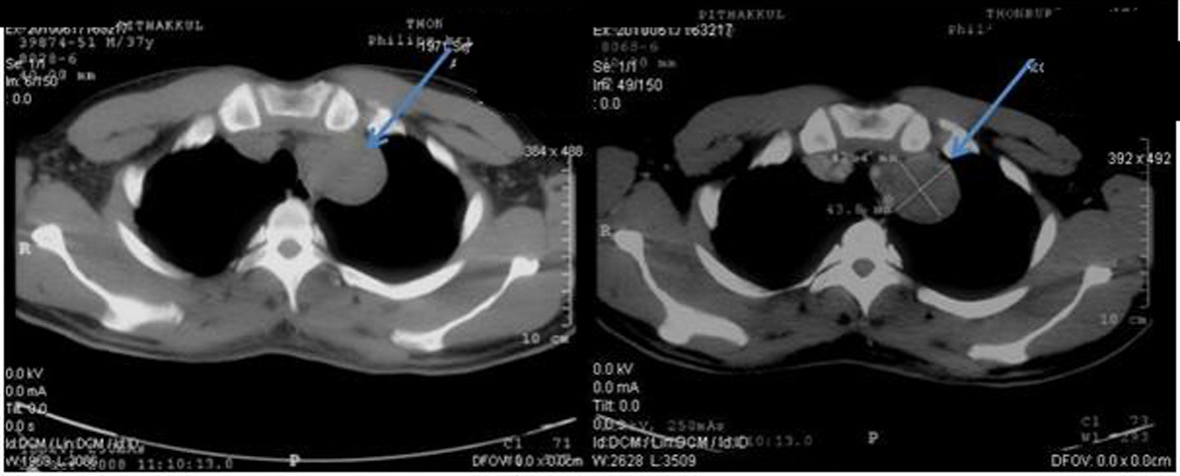
Pretreatment CT scan of chest in October 2008 (Pre-contrast on left side and post-contrast on right side) showing lobulated heterogeneous soft tissue density lesion at the apex of left lung.

**Figure 2:**
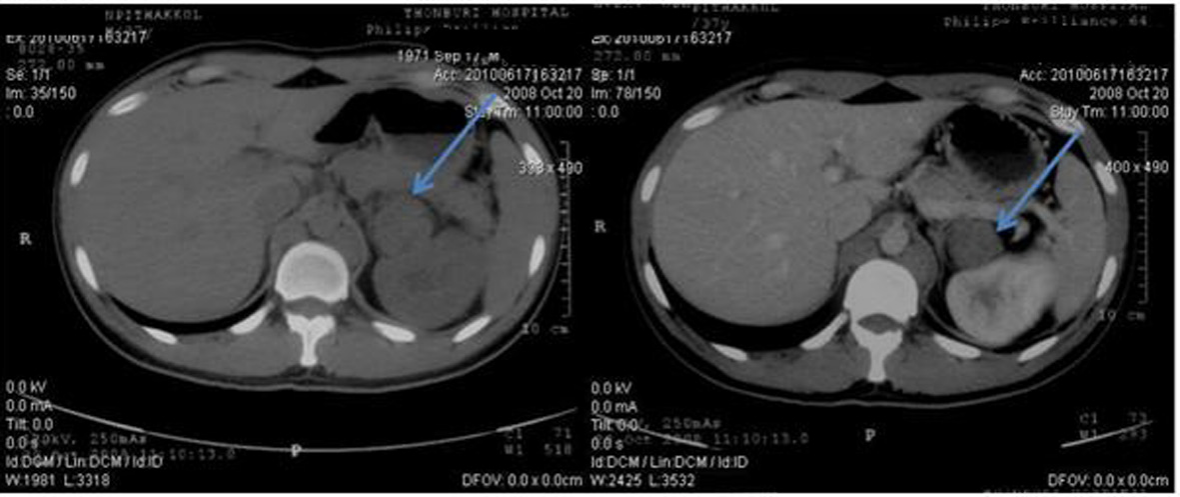
Pretreatment CT scan of abdomen in October 2008 (Pre-contrast on left side and post-contrast on right side) showing paraaortic lymph nodes enlargement on left side partially abuts wall of aorta and body of pancreas.

**Figure 3:**
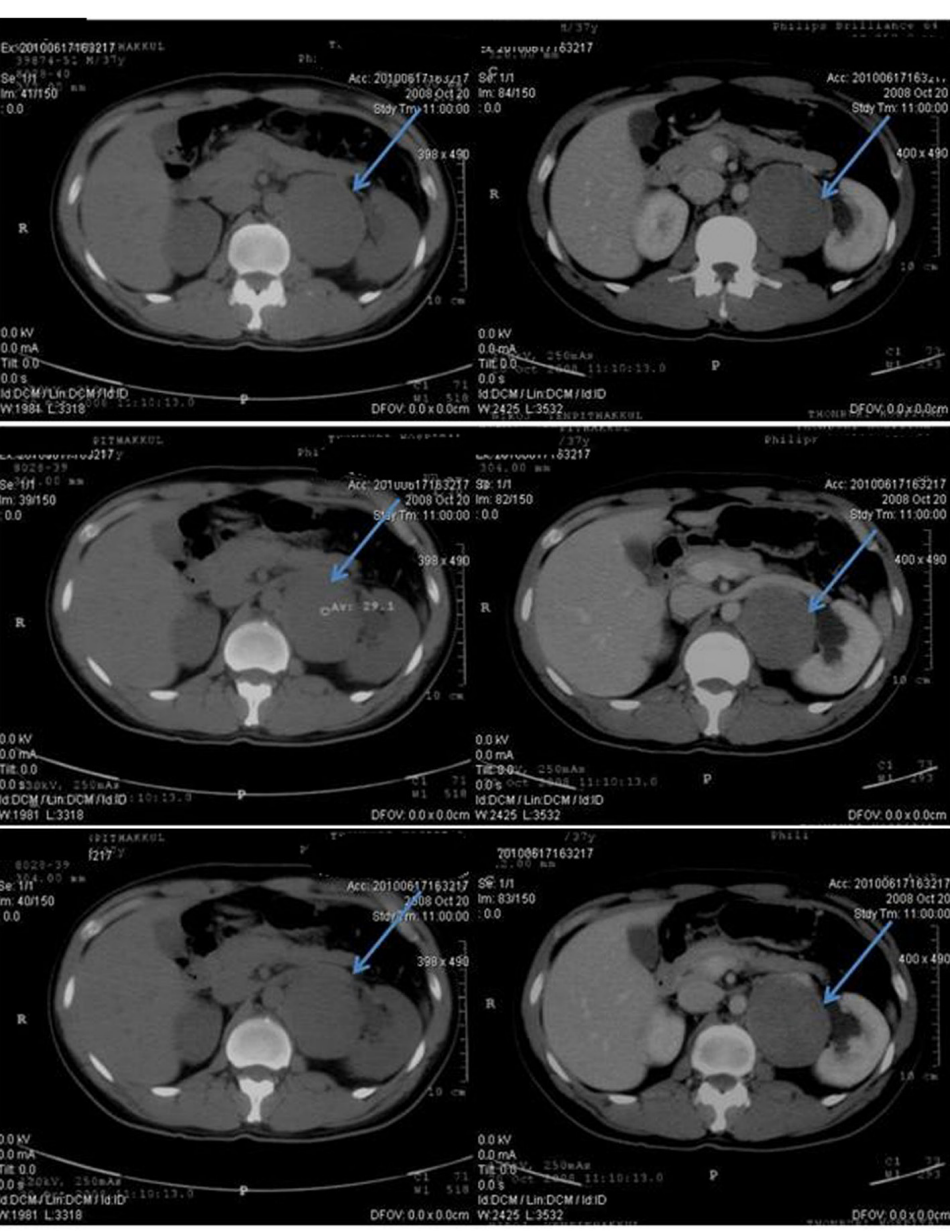
Pretreatment CT scan of abdomen in October 2008 (Pre-contrast on left side and post-contrast on right side) showing enlargement of multilobulated left paraaortic node at the renal level, 7.4 cm in size. The mass displaces the left renal vein anteriorly. Some mass effect is noted with mildly dilated left renal pelvis.

In October 2008, a 37-year-old previously healthy Thai male, with 3cm left supraclavicular lymph node enlargement. The lymph node was biopsied and sent to pathologist and the result showed metastatic well differentiated adenocarcinoma. CT chest and abdomen was done to search for the primary site. It revealed mildly reticular densities at right lung apex, malignant lobulated heterogeneous soft tissue mass at left neck extending downward to level of the aortic arch. Direct tumor invasion was seen at left common carotid and subclavian arteries and left lobe of thyroid gland and partially abuts wall of esophagus. Left paraaortic multi-lobulated nodes were enlarged at the renal level. The mass displaced the left renal vein anteriorly. Some mass effect was noted with mildly dilated left renal pelvis. The 7.4 cm lesion partially abutted wall the aorta and body of the pancreas (Fig. 1-3).Tumor markers including AFP, LDH and CEA were within normal limit.

**Figure 4:**
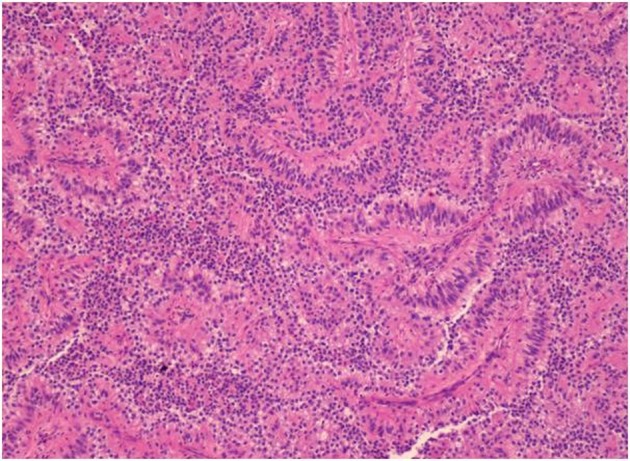
Hematoxylin & Eosin stain (H&E stain) of biopsied left supraclavicular lymph node showing lymph node with metastatic epithelial neoplasm arranging in papillary growth pattern. The neoplastic cells have enlarged pleomorphic and prominent nuclei, eosinophilic and clear neoplasm.

At that time, preliminary diagnosis was metastatic adenocarcinoma of lung cancer as the primary site. The physician started treatment in October 2008 with 2 cycles of palliative chemotherapy, carboplatin and paclitaxel. However, the disease progressed. CT chest and abdomen in January 2009 revealed left superior mediastinal node slightly increased and the mass at the pararenal region and necrotic node, paravertebral node were about the same. Treatment was switched to 2nd line with cisplatin and Etoposide for 2 cycles. After the 2nd cycle, he lost follow up for 1 year because of chemotherapy side effect. A year later, the patient went to Chulabhorn hospital for a second opinion. The pathology of lymph node was evaluated and formed to be metastatic papillary adenocarcinoma (Fig. 4). Further immunohistochemistry for TTF-1, Thyroglobulin, CD7 and CD20 were all negative.

CD10 AE1/AE3, Vimentin and RCC were positive, suggesting that the disease was renal cell carcinoma. Therefore, the patient was diagnosed with papillary cell type metastatic renal cell carcinoma without renal mass as primary site. In the present time, the patient is doing well with stable disease and is being treated with Sunitinib cycle15th (last September 2012). Follow-up CT scan still demonstrated no primary kidney lesion.

## Discussion

In this case report, there are interesting aspects to be discussed. First of all is the clinical manifestation of the patient. He presented with multiple sites of metastatic lymph nodes without primary mass at kidney which is very rare condition to be found. Although, renal cell carcinoma is well-known for its ability to metastasize to almost every organ in the body, most of the cases presented with renal mass. We reviewed previously published case report and found only two case reports which were renal cell carcinoma without renal primary lesion. The first one presented with pancreatic, parotid and subcutaneous mass [3]. The difference between the mentioned case report and this one is the histology of the cancer. The published one is clear cell which was the most common cell type of renal cell carcinoma but this one is papillary renal cell carcinoma. The second case was also papillary renal cell carcinoma case who presented with metastasis of lymph nodes similar to this one [4].

Secondly, since the patient was presented with cancer of unknown origin. The approach to unknown primary site cancer should be discussed here. From the supraclavicular lymph node pathology result, papillary cell adenocarcinoma, the differential diagnoses were papillary thyroid cancer, gastrointestinal tract carcinoma such as pancreato-biliary cancer, non small cell lung cancer, and renal cancer. The immunohistochemistry should be done to differentiate the origin of tumor. TTF-1, Thyroglobulin, CD7 and CD20 were tested to rule out non-small cell lung adenocarcinoma, thyroid cancer and gastrointestinal tract cancer respectively. They were all negative. Further immunohistochemistry tests including CD10, Vimentin and RCC were all positive and all were specific for renal cell carcinoma. Typically, CD10 is positive in 67-93% of papillary cell renal cancer. Still, CD10 is also positive in pheochromocytoma. Vimentin and RCC marker was therefore tested for the diagnosis. Vimentin is expressed in most (87-100%) clear cell and papillary renal cell carcinoma. The renal cell carcinoma marker is positive in almost all papillary and clear cell renal cell carcinoma (87-95% and 72-85%) but is uniformly negative for collecting duct renal cell carcinoma and oncocytoma [5]. To summarize, immunohistochemistry plays an important role in the diagnosis of cancer of unknown origin and renal cell carcinoma should be considered as one of the differential diagnosis in papillary cell type cancer.

Lastly, treatment option for papillary cell renal cancer should be raised here. The diagnosis is metastatic papillary renal cell carcinoma. From MSKCC criteria 2002, the patient was categorized in favorable risk group. Thus, he is being treated with Sunitinib, a class of tyrosine kinase inhibitor, which is a novel targeted therapy.

Generally, renal cell carcinoma is resistant to chemotherapy or radiation. Prior to Sunitinib, metastatic disease could only be treated with the IFNα or interleukin-2. However, these agents demonstrated low rates of efficacy (5-20%). Sunitinib shows longer median progression free survival than IFNα (11 months vs 5 months, P < 0.000001). Objective response rate is also superior (39-47% for sunitinib versus 8-12% with IFNα, P < 0.000001). And patients in the sunitinib group had a significantly better quality of life than patients in the interferon alfa group (P < 0.001). Although most of subjects in the studies are clear cell type RCC, subgroup analysis also demonstrated better result in papillary cell type RCC [6-8]. Sunitinib also shows satisfactory response in this patient. He has been treated for almost 2 years (October 2010 - September 2012) with stability of disease.

### Conclusion

This case presents metastatic papillary renal cell carcinoma without renal lesion as primary site with the first clinical manifestation as cancer of unknown origin with lymph node enlargement. The diagnosis was made on the basis of histology which showed papillary cell type. In order to narrow the possibility, immunohistochemistry is very helpful and leads to further proper management.
